# Social networking site addiction and undergraduate students’ irrational procrastination: The mediating role of social networking site fatigue and the moderating role of effortful control

**DOI:** 10.1371/journal.pone.0208162

**Published:** 2018-12-11

**Authors:** Shuai-lei Lian, Xiao-jun Sun, Zong-kui Zhou, Cui-ying Fan, Geng-feng Niu, Qing-qi Liu

**Affiliations:** 1 Key Laboratory of Adolescent Cyberpsychology and Behavior (CCNU), Ministry of Education, Wuhan, China; 2 School of Psychology, Central China Normal University, Wuhan, China; 3 Institute of Social Psychology, School of Humanities and Social Science, Xi’an Jiaotong University, Xi’an, China; University of Colorado Boulder, UNITED STATES

## Abstract

With the popularity of social networking sites (SNSs), the problems of SNS addiction have been increasing. Research has revealed the association between SNS addiction and irrational procrastination. However, the mechanism underlying this relation is still unclear. The present study aimed to examine the mediating role of social networking site fatigue and the moderating role of effortful control in this link among Chinese undergraduate students. The Social Networking Site Addiction Scale, Social Networking Service Fatigue Scale, Effortful Control Scale and Irrational Procrastination Scale were completed by 1,085 Chinese undergraduate students. Results indicated that SNS addiction, SNS fatigue and irrational procrastination were positively correlated with each other, and negatively correlated with effortful control. Further analyses revealed that, SNS addiction has a direct effect on irrational procrastination. SNS fatigue mediated the relationship between SNS addiction and irrational procrastination. Both direct and indirect effects of SNS addiction on irrational procrastination were moderated by effortful control. Specifically, this effect was stronger for people with lower effortful control. These findings help clarify the mechanism underlying the association between SNS addiction and irrational procrastination, which have potential implications for intervention.

## Introduction

Social networking sites (SNSs) provide a platform for users to interact with each other by sharing personal information and pictures and getting feedback from other users [1]. These internet platforms have become increasingly popular among undergraduate students [2]. With the spreading of SNSs, SNSs have become the essential element of undergraduate students’ everyday life [3, 4]. What is noteworthy is that, SNS is a double-edged sword, which brings people such a great convenience on the one hand, and causes psychological problems on the other hand, such as SNS addiction, procrastination and depressive symptoms [5–7]. Especially, SNS addiction has become a widespread concern of researchers. Therefore, numerous studies have examined factors that influence SNS addiction as well as the effects of SNS addiction on students’ life satisfaction and mental health [8, 9].

SNS addiction is a term that coexists with other terms (e.g., “problematic SNS use”, “SNS addiction”, “SNS intrusion”) referring to a fundamentally similar concept of SNS use as a potential behavioral addiction [10]. The term “problematic SNS use” lays stress on the preference for online social interaction and the term “SNS intrusion” emphasizes the aspect of relations with others [11, 12]. “SNS addiction” is defined as a specific form of internet addiction applicable to individuals who are excessively involved in SNS activities, and thus experiencing detrimental effects on their lives [4, 6]. It is characterized by six typical characteristics: (1) salience (e.g., activities on SNSs dominate thinking and behavior); (2) withdrawal symptoms (e.g., experiencing unpleasant feelings when the activities on SNS are discontinued or suddenly reduced); (3) relapse (e.g., a tendency to revert to earlier patterns of SNS use after ineffective abstinence or control); (4) mood modification (e.g., activities on SNS modifies or improves mood); (5) tolerance (e.g., increasing time on SNS is required to achieve previous using effects); and (6) conflict (e.g., conflicts in relationships and other activities caused by intensive use of SNS) [13–15].Compared to “problematic SNS use” and “SNS intrusion”, SNS addiction puts more emphasis on the detrimental effects of excessive SNS use on everyday social functioning [6]. In the present study, excessive SNS use is considered as the primary cause of irrational procrastination. Therefore, SNS addiction is defined as excessive involvement in SNS activities and a frequent cause of social and personal problems in everyday lives.

Students with SNS addiction are overly concerned about SNSs driven by a strong motivation to log on to or use SNSs and devote much time and effort to SNSs [16]. SNS addiction has been proved to have adverse effects on other social activities, studies/job, interpersonal relationships, and/or psychological health and well-being [16]. Previous research also indicated that undergraduate students suffering from SNS addiction may regard SNS as a tool for procrastination [7]. Procrastination, in turn, has various negative consequences for individuals, including higher stress, more depression, anxiety, fatigue and lower satisfaction across life domains [17]. Therefore, procrastination is an important dependent variable to study in the context of SNS addiction.

Procrastination has been specified into three types: decisional procrastination (inability to make a decision within a specified time period); arousal procrastination (purposefully waiting until the last minute for a thrill-seeking sensation, yielding pleasure from “beating the clock”); and avoidance procrastination (delayed motivation by a desire to prevent performance evaluation and fears) [18]. However, a meta-analytic review conducted by Steel showed that previous empirical research could not provide support for this trinity of procrastination scales, especially regarding avoidant procrastination and arousal procrastination [19]. Instead, the results indicated that procrastination is predominantly considered as an irrational procrastination [19]. Irrational procrastination refers to the intentional delay of action despite knowing that one will be worse off due to the delay [20, 21]. According to the standpoints of some researchers, although irrational procrastination can be treated as a relatively stable personality trait caused by self-regulatory failure, it also could be predicted by factors such as low conscientiousness, high impulsivity, negative affect and the lack of self-control [20, 22, 23].

With the increasing prevalence of SNS addiction, it has been recognized as a new predictor of irrational procrastination based on the following reasons. First, individuals with SNS addiction access SNS more frequently for entertainment purposes and pay continuous attention to checking SNS for news and messages from friends [24]. SNS checking habit and SNS enjoyment may drive users to procrastinate with their subjectively aversive tasks such as writing a term paper [7]. Second, SNS addiction or SNS use has been shown to be positively associated with various mental health problems, such as symptoms of depression, anxiety, and stress [9,13, 25]. These mental health problems have been further revealed as the significant causes of irrational procrastination [20, 23]. Moreover, empirical research also demonstrated that SNS has been took as a particularly prominent tool for procrastination among students [7]. Therefore, undergraduate students suffering from SNS addiction may be more likely to procrastinate irrationally. Based on the above analyses, we put forward the following hypothesis:

**Hypothesis 1a.** SNS addiction will be positively related to irrational procrastination.

Although research has revealed a positive association between SNS addiction and irrational procrastination, the mechanisms underlying this association is still unclear. In other words, it remains unclear how (or why), and when (i.e., under what conditions) SNS addiction predicts irrational procrastination. Absent understanding of the mechanisms connecting SNS addiction to irrational procrastination, research can offer only limited practical guidance for undergraduate students as well as for university educators to develop intervention strategies. To fill this gap, researchers need to take intervening and contextual factors into account when revealing the relationship between SNS addiction and irrational procrastination. First, researchers need to explicate the factors that carry the influence of SNS addiction on irrational procrastination (mediation). Second, researchers need to explore the contextual factors on which the direct and indirect effects of SNS addiction on irrational procrastination depend (moderation). Therefore, the present study, by constructing a moderated mediation model, attempted to open the black boxes of the mechanisms that explain in greater detail how (or why) and when SNS addiction can lead to irrational procrastination. Specifically, this study examined the mediating effect of SNS fatigue and the moderating effect of effortful control in the association between SNS addiction and irrational procrastination. This integrated model could not only answer the question of how SNS addiction affects irrational procrastination, but also provide valuable information for us to understand when SNS addiction affects irrational procrastination the most.

### SNS fatigue as a mediator

Fatigue refers to a subjective feeling of discomfort, decreased motivation, and increased physical lassitude and task aversion [26]. In the current information society, the association between excessive SNS use and fatigue is attracting more and more attention [26, 27]. The concept of SNS fatigue was proposed to describe the subjective feeling of tiredness from excessive SNS use [26]. It is a form of strain caused by information and communication technology overload in SNS [26]. Remarkably, although “SNS fatigue” is similar to “social media fatigue” literally, they are two completely different concepts. SNS fatigue emphasizes the feeling of being extremely tired after being involved in SNS activities for a long time [26]. Social media fatigue emphasizes the feeling of not wanting to use social media [28]. In the present study, SNS fatigue was considered as a subjective and self-evaluated feeling of being exhausted by excessive SNS use which may lead to social and personal problems, such as irrational procrastination. SNS fatigue has been found to be positively correlated with irrational procrastination [26, 27, 29]. This relation is in agreement with the assumption of self-regulation failure theory, which argues that irrational procrastination is essentially a self-regulatory failure [30].People who experience fatigue are also thought to have depleted resources and to be less capable of adequate self-regulation [29].

SNS fatigue can be induced by many factors, such as information or communication technology overload, low self-esteem, poor sleep quality, and the interactions among these factors [27, 29]. SNS addiction may be another important factor that can lead to SNS fatigue. Although the direct effect of SNS addiction on SNS fatigue has not been demonstrated in previous research, three reasons could be used to argue for it.

Firstly, individuals experiencing SNS addiction are more likely to pay continuous attention to SNS in order to respond to or get feedback from others' message or postings in a timely fashion [31]. They may be impeded by the overwhelming volume of information and social demand, which may drive them to their cognitive limits and leave them feeling overwhelmed, exhausted and SNS fatigue [26,32]. Thus, SNS fatigue may be induced by “overload” and “excessive social demands” resulting from SNS addiction. Secondly, social networking sites are awash with information with a positive bias [33]. SNS use is essentially a process of long-term exposure to positive information and upward social comparisons for individuals experiencing SNS addiction [34]. According to social comparison theory and empirical research, negative affect occurs after experiencing upward social comparison [5, 35]. Cramer and colleagues provided empirical evidence for the positive link between motives for social comparison on Facebook and Facebook fatigue [27]. Thus, SNS addiction may induce and exacerbate SNS fatigue due to the high frequency of upward social comparison on SNS. Thirdly, individuals who have developed dependence on SNS spend a great amount of time using them in bed and have less sleep hours and poorer sleep quality due to late night logins [36, 37], which has been proved to be positively associated with fatigue [38]. Given that fatigue is positively related to irrational procrastination [29], it is reasonable to assume that SNS addiction increases SNS fatigue, which in turn results in irrational procrastination. We put forward the following hypothesis:

**Hypothesis 1b.** SNS fatigue will mediate the positive relation between SNS addiction and irrational procrastination.

### Effortful control as a moderator

Although SNS addiction may lead to SNS fatigue and irrational procrastination, it is possible that not all individuals experiencing SNS addiction show these outcomes to the same degree. It is necessary to examine individual variables that may play a buffering role in the process by which SNS addiction induces negative outcomes. In the present study, effortful control was tested as a moderator. Specifically, we examined whether the direct effect of SNS addiction on irrational procrastination and the indirect effect via SNS fatigue would be moderated by effortful control.

Effortful control is the core component of self-regulation and refers to an individual’s ability to actively modulate physiological, emotional, and behavioral responses [39, 40]. Effortful control is positively associated with social adaptation [41, 42], academic competence and achievement [43, 44], while being negatively associated with internalizing and externalizing behavior problems [41, 42]. According to Steel, procrastination is a prevalent and pernicious form of self-regulatory failure, which could be seen as the direct consequence of low effortful control [20]. Research also demonstrated that attention control, as one of the core components of effortful control, has turned out to be negatively correlated with procrastination [45].

Effortful control emerges in the context of the social environment during childhood and shows different levels among different people [46]. It also has been found to play an important buffering role in the process of risk factors leading to internal and external problems. For instance, Lengua and colleagues found higher effortful control buffered the adverse impact of risk factors on children, allowing them to deal best with the adverse experience and avoid internalizing and externalizing problems [39].A study by Bao and colleagues showed that the inhibitory effect of a supportive school climate on adolescent delinquency was stronger for individuals with higher effortful control [47].Another study indicated that the relation between perceived school climate and deviant peer affiliation was more potent for individuals with lower effortful control [48].

Therefore, effortful control, as a risk-buffering factor, may mitigate the potential adverse outcomes (e.g., SNS fatigue and irrational procrastination) of SNS addiction. In other words, effortful control may buffer the negative effects of SNS addiction on SNS fatigue and irrational procrastination. In addition, given that people with high effortful control are better at handling the state of exhaustion and attenuating negative emotions [49, 50], high levels of effortful control might serve as a protective factor to reduce SNS fatigue as a potential risk factor for irrational procrastination. Therefore, effortful control may play a moderating role in the relationship between SNS fatigue and irrational procrastination. Specifically, both the direct effect of SNS addiction on irrational procrastination and the underlying indirect effect of SNS fatigue may be moderated by effortful control.

Therefore, we put forward the following hypotheses:

**Hypothesis 2.** Effortful control would moderate the relation between SNS addiction and irrational procrastination, with the relation being stronger for students with lower effortful control.

**Hypothesis 3.** Effortful control would moderate the mediating effect of SNS fatigue in the relation between SNS addiction and irrational procrastination, with the mediating effect of SNS fatigue being stronger for students with lower effortful control.

### The present study

**Fig 1:**
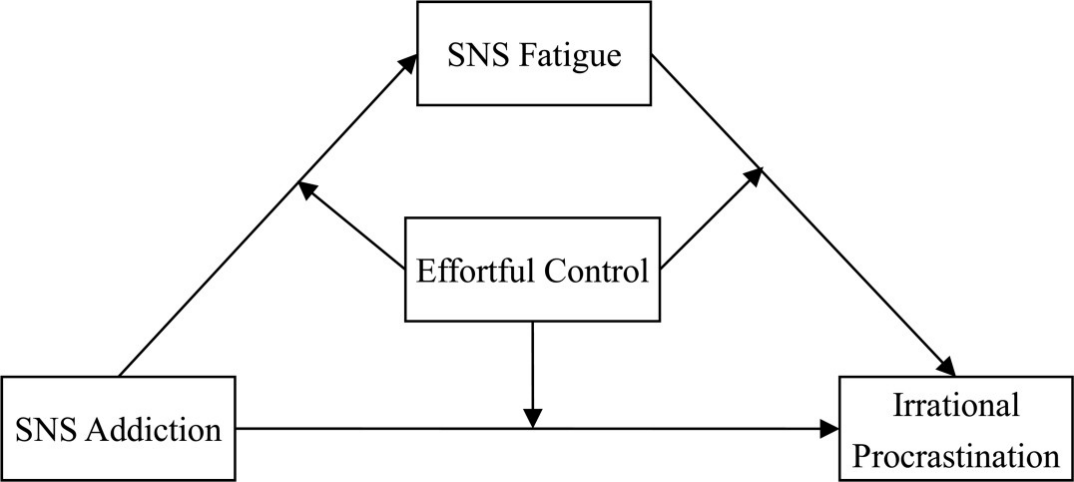
Conceptual model.

This study examined the potential mechanism underlying the association between SNS addiction and irrational procrastination among Chinese undergraduate students. A moderated mediating model (see Fig 1) was constructed to investigate this question. To test both mediator and moderator variables in a single model can provide more insightful knowledge about the relationship between the two variables [51]. The mediator variable in this model explains how the predictor is linked to the criterion. The moderator variable in this model answers when the direct and indirect relations between the predictor and the criterion are tenable. In the present study, SNS fatigue was considered as a mediator and effortful control was treated as such a moderator.

## Method

### Participants

A total of 1,085 students (57.1% female) were recruited from two universities in two major cities in China (Hangzhou and Wuhan) using convenience sampling. Three hundred and forty-seven (32.0%) of them are 1st-year students; three hundred and seventy-six (34.7%) of them are 2nd-year students; and three hundred and sixty-two (33.3%) of them are 3rd-year students. The age of the participants varied from 18 to 24 years, with a mean age of 19.66 years (SD = 1.16).

### Procedure

Convenience sampling was adopted to choose three to four classes in each grade from 1st-year students to 3rd-year students at each of the two target universities. Students in selected classes were invited to participate in this study if they were SNS users. Questionnaires were designed to collect information including demographic variables, SNS addiction, irrational procrastination, SNS fatigue, and effortful control. Participants were informed of the requirements of this study by using standard instructions, emphasizing the authenticity, independence, and integrity of all answers. They were also informed that all of their answers would be kept confidential. The survey was conducted as paper-and-pencil in different classrooms taking a class as a unit. All of the questionnaires were completed anonymously in 45 minutes after informed consent was obtained from the schools, teachers, and participants. This study was approved by the Ethical Committee for Scientific Research at Central China Normal University, and a signed consent form was collected from each student's parents.

### Measurements

#### Social networking site addiction

Social networking site addiction was assessed using the revised version of the Facebook Addiction Scale (FAS) [4]. In order to enhance the applicability of the FAS for Chinese undergraduate students, we replaced the word "Facebook" in the original questionnaire with "social networking sites", and translated every item into Chinese. This scale includes eight items and assesses factors related to social networking site addiction, including the symptoms of cognitive and behavioral salience, conflict with other activities, euphoria, relapse and reinstatement, withdrawal, and loss of control (e.g., I feel anxious if I cannot access to Facebook). Participants responded on a Likert-type scale ranging from 1 (not true) to 5 (extremely true). Responses were averaged to form a measure of undergraduate students’ SNS addiction. Same as the structure of the original scale, the Chinese version of the Social Networking Site Addiction Scale was one-dimensional structure. The confirmatory factor analysis (CFA) indexes generated by Amos 21.0 showed that this one-dimensional measurement model had a good fit with the data: χ2/df = 4.43, RMSEA = 0.06, CFI = 0.98, NFI = 0.97, GFI = 0.98. The items also demonstrated high reliability in the present study (Cronbach’s α = 0.86).

#### Irrational procrastination

Irrational procrastination was assessed using the Chinese version of the Irrational Procrastination Scale, which was translated from the English version of the Irrational Procrastination Scale (IPS) [52]. Participants responded to the nine items assessing the degree of irrational delay causing procrastination on a Likert-type scale ranging from 1 (strongly disagree) to 5 (strongly agree) (e.g., My life would be better if I did some activities or tasks earlier). Same as the structure of the original scale, the Chinese version of the Irrational Procrastination Scale was one-dimensional structure. The CFA indexes generated by Amos 21.0 showed that this one-dimensional measurement model had a good fit with the data: χ2/df = 4.35, RMSEA = 0.06, CFI = 0.98, NFI = 0.97, GFI = 0.99.The items also demonstrated acceptable reliability in the present study (Cronbach'sα = 0.73).

#### Social networking site fatigue

The degree of SNS users’ subjective feelings of tiredness from SNS use was assessed using the Chinese version of Social Networking Site Fatigue Scale, which was translated from the English version of Social Networking Site Fatigue Scale (SFG) [26]. This scale consists of five items, all of which were adapted from Yperenand Hagedoorn [53]. Participants responded to these items on a Likert-type scale ranging from 1 (strongly disagree) to 7 (strongly agree) (e.g., Due to using SNSs, I feel rather exhausted). Responses were averaged to form a measure of fatigue from SNS. Same as the structure of the original scale, the Chinese version of the Chinese version of Social Networking Site Fatigue Scale was one-dimensional structure. The CFA indexes generated by Amos 21.0 showed that this one-dimensional measurement model had a good fit with the data: χ2/df = 3.82, RMSEA = 0.05, CFI = 0.99, NFI = 0.99, GFI = 0.99. The items also demonstrated high reliability in the present study (Cronbach'sα = 0.84).

#### Effortful control

Effortful control was assessed using the Effortful Control Scale, a Chinese-language measure developed by Li, Zhang, Li, Zhen and Wang (2010) [54]. Participants responded tothe16 items on a Likert-type scale ranging from 0 (never) to 6 (always) (e.g., Even if I know that I shouldn’t do those things, I still do them anyway). Responses (with negative items being reverse coded) were averaged to form a measure of students’ effortful control, with higher scores representing higher levels of effortful control. This scale has been used in a sample of Chinese university students with good reliability and validity [49]. In the current study, the items demonstrated acceptable reliability (Cronbach’sα = 0.74).

### Control variables

According to prior research on SNS addiction [55], there were marked difference among the surveyed adolescents of different genders and grades in SNS addiction. Andreassen, Torsheim, Brunborg, and Pallesen also suggested that SNS addiction was correlated with age, with the prevalence being higher among younger people [14]. Thus, gender, age and grade were chosen as the control variables in this study.

### Statistical analyses

Firstly, descriptive statistics, Pearson correlations, and two-way MANOVA analyses for gender and grade were conducted to examine the range, means, standard deviations, bivariate associations, and variance for all research variables using SPSS 23.0. Secondly, the SPSS macro PROCESS (model 59; http://www.afhayes.com) suggested by Hayes (2013) was used to test the proposed moderated mediation model [56]. Because it is able to test mediating models, moderating models, moderated mediating models, and other complex models, this SPSS macro has been used by several researchers [57–60]. Thirdly, simple slopes analyses were performed to decompose all significant interaction effects [61].

## Results

### Preliminary analyses

The results for the two-way MANOVA indicated a significant main effect for gender (Wilks' lambda = 0.977, F (4, 1076) = 6.267, p <0.01) and a significant interaction between grade and gender (Wilks' lambda = 0.983, F (8, 2152) = 2.292, p <0.05). Because the interaction between grade and gender was significant, one-way MANOVA was conducted to examine the gender differences for each of the three grade levels separately. The results for 1st-year students showed that irrational procrastination and SNS fatigue showed no gender differences, whereas both SNS addiction (F (1, 345) = 4.602; p <0.05) and effortful control (F (1, 345) = 5.567; p <0.05) showed significant gender differences. SNS addiction was significantly greater for females (M = 2.69) than for males (M = 2.50), whereas effortful control was significantly higher for males (M = 3.78) than for females (M = 3.62). The results for 2nd-year students showed that irrational procrastination and SNS fatigue showed no gender differences, whereas both SNS addiction (F (1, 374) = 5.245; p< 0.05) and effortful control (F (1, 374) = 15.510; p <0.001) showed significant gender differences. SNS addiction was significantly higher for females (M = 2.74) than for males (M = 2.56), whereas effortful control was significantly higher for males (M = 3.75) than for females (M = 3.55). The results for 3rd-year students showed that effortful control and SNS fatigue showed no gender differences, whereas both SNS addiction (F (1, 360) = 9.635; p <0.01) and irrational procrastination (F (1, 360) = 7.540; p <0.01) showed significant gender differences. Both SNS addiction and irrational procrastination (IP) were significantly higher for females (M_SNS addiction_ = 2.85; M_IP_ = 3.43) than for males (M_SNS addiction_ = 2.60; M_IP_ = 3.27). None of the observed variables showed a significant correlation with age. However, because of prior evidence suggesting age differences in SNS addiction, we included age as a control variable, along with gender and grade, as planned.

Table 1 presents the means, standard deviations, and correlations for all of the observed variables and age. As hypothesized, SNS addiction was positively correlated with SNS fatigue and irrational procrastination and negatively correlated with effortful control. SNS fatigue was positively correlated with irrational procrastination and negatively correlated with effortful control. Effortful control was negatively correlated with irrational procrastination.

**Table 1:** Descriptive statistics and interrelations among all of the observed variables and age.

Variables	M	SD	1	2	3	4	5
1.SNS addiction	2.67	0.76	1.00				
2. SNS fatigue	3.51	1.16	0.45**	1.00			
3. Irrational procrastination	3.33	0.57	0.33**	0.32**	1.00		
4. Effortful control	3.65	0.55	-0.34**	-0.17**	-0.19**	1	
5. Age	19.66	1.16	0.02	0.02	-0.02	-0.03	1

Note. N = 1085.

**p< 0.01.

### Testing for the proposed moderated mediation model

Before testing the proposed moderated mediation model, we tested the mediation model firstly. Table 2 presents the main results generated by Hayes’s (2013) SPSS macro PROCESS. It includes total effect model, mediator and dependent variable model, and specific effect analysis (including total effect, direct effect and indirect effect). The total effect model was employed to test the total effect of SNS addiction on irrational procrastination. The mediator variable model was employed to test the effect of SNS addiction on SNS fatigue. The dependent variable model was employed to test the effects of SNS addiction and SNS fatigue on irrational procrastination. Specific effect analysis was employed to examine the significance of the total effect, direct effect and indirect effect and the ratio of direct effect and indirect effect to the total effect.

**Table 2:** Regression results for the mediation model.

Model									
Model 1: Total effect model									
R	R^2^	F	df_1_	df_2_	p	B	SE	t	p
0.33	0.11	35.21	4	1080	< 0.001				
Constant						3.56	0.40	9.01***	< 0.001
Gender						-0.03	0.03	-0.84	>0.05
Age						-0.05	0.02	-2.27*	<0.05
Grade						0.06	0.03	1.03*	<0.05
SNS addiction						0.25	0.02	11.59***	< 0.001
Model 2: Mediator variable model									
R	R^2^	F	df_1_	df_2_	p	B	SE	t	p
0.45	0.20	56.64	4	1080	< 0.001				
Constant						0.43	0.78	0.55	>0.05
Gender						0.05	0.07	0.77	>0.05
Age						0.07	0.04	1.61	>0.05
Grade						-0.10	0.06	-1.60	>0.05
SNS addiction						0.68	0.05	14.54***	< 0.001
Model 3: Dependent variable model									
R	R^2^	F	df_1_	df_2_	p	B	SE	T	p
0.39	0.15	38.50	5	1079	< 0.001				
Constant						3.51	0.39	8.97***	< 0.001
Gender						-0.03	0.03	-1.03	>0.05
Age						-0.06	0.02	-2.65**	< 0.01
Grade						0.07	0.03	2.44*	< 0.05
SNS addiction						0.17	0.02	7.83***	< 0.001
SNS fatigue						0.11	0.01	7.61***	< 0.001
Specific effect analysis									
		B		SE	LLCI	ULCI	Ratio of direct effect or indirect effect to total effect		
Total effect		0.25		0.02	0.20	0.29			
Direct effect		0.17		0.02	0.13	0.21	68%		
Indirect effect		0.08		0.01	0.06	0.10	32%		

Note. N = 1085. Unstandardized regression coefficients are reported. Bootstrap sample size = 5000. LL = low limit, CI = confidence interval, UL = upper limit.

*p< 0.05.

**p< 0.01.

***p< 0.001.

As can be seen from total effect model (F(4, 1080) = 35.21, R^2^ = 0.11, p <0.001), after controlling for gender, age, and grade, SNS addiction had a significant positive total effect on irrational procrastination(B = 0.25, p <0.001). The mediator variable model (F(4, 1080) = 56.64, R^2^ = 0.20, p <0.001) and the dependent variable model (F (5, 1079) = 38.50, R^2^ = 0.15, p <0.001) showed that, after controlling for gender, age, and grade, SNS addiction positively predicted SNS fatigue (B = 0.68, p <0.001), SNS fatigue positively predicted irrational procrastination (B = 0.11, p <0.001), and SNS addiction positively predicted irrational procrastination (B = 0.17, p <0.001). The specific effect analysis indicated that all of the total effect, direct effects and indirect effects were positively and significantly different from zero. The direct effect and indirect effect accounted for 68% and 32% of the total effect, respectively.

Base on the testing for the mediation model, we used Hayes’s (2013) SPSS macro PROCESS to test the proposed moderated mediation model.

Table 3 presents the main results of testing for the proposed moderated mediation model. It includes mediator and dependent variable model, conditional direct effect analysis and conditional indirect effect analysis. The mediator variable model was employed to test the effects of SNS addiction and effortful control on SNS fatigue. The dependent variable model was employed to test the effects of SNS addiction, SNS fatigue, and effortful control on irrational procrastination. Conditional direct effect analysis was conducted to test the changes in variance explained by the direct effect of SNS addiction on irrational procrastination as a function of an increase in individuals’ effortful control. Conditional indirect effect analysis was conducted to test the changes in the variance explained by the indirect effect of SNS fatigue in the link between SNS addiction and irrational procrastination as a function of an increase in individuals’ effortful control.

**Table 3:** Regression results for the conditional indirect effects (moderated mediation).

Model									
Model 1: Mediator variable model									
R	R^2^	F	df_1_	df_2_	p	B	SE	t	p
0.46	0.21	41.02	6	1078	< 0.001				
Constant						-1.27	0.78	-1.62	>0.05
Gender						0.05	0.07	0.72	>0.05
Age						0.07	0.04	1.58	>0.05
Grade						-0.10	0.06	-1.57	>0.05
SNS addiction						0.66***	0.05	13.54	< 0.001
Effortful control						-0.04	0.06	-0.61	>0.05
SNS addiction × Effortful control						-0.26***	0.07	-3.56	< 0.001
Model 2: Dependent variable model									
R	R^2^	F	df_1_	df_2_	p	B	SE	t	p
0.41	0.17	31.63	8	1076	< 0.001				
Constant						4.37***	0.39	11.21	< 0.001
Gender						-0.04	0.03	-1.18	>0.05
Age						-0.06**	0.02	-2.70	< 0.01
Grade						0.07*	0.03	2.39	< 0.05
SNS addiction						0.14***	0.02	6.35	< 0.001
SNS fatigue						0.10***	0.01	7.05	< 0.001
Effortful control						-0.09***	0.02	-3.90	< 0.001
SNS addiction × Effortful control						-0.07*	0.03	-2.48	< 0.05
SNS fatigue × Effortful control						-0.08***	0.02	-3.79	< 0.001
Conditional direct effect analysis at values of effortful control (M ± SD)									
						B	SE	LLCI	ULCI
M– 1SD (3.09)						0.18	0.03	0.12	0.23
M (3.65)						0.14	0.02	0.10	0.18
M + 1SD (4.20)						0.10	0.02	0.05	0.15
Conditional indirect effect analysis at values of effortful control (M ± SD)									
						B	Boot SE	BootLLCI	BootULCI
M– 1SD (3.09)						0.12	0.02	0.09	0.16
M (3.65)						0.07	0.01	0.05	0.09
M + 1SD (4.20)						0.03	0.01	0.01	0.05

Note. N = 1085. Unstandardized regression coefficients are reported. Bootstrap sample size = 5000. LL = low limit, CI = confidence interval, UL = upper limit.

*p< 0.05.

**p< 0.01.

***p< 0.001.

As can be seen from mediator variable model (F(6, 1078) = 41.02, R^2^ = 0.21, p <0.001) and the dependent variable model (F (8, 1076) = 31.63, R^2^ = 0.17, p <0.001), after controlling for gender, age, and grade, SNS addiction positively predicted SNS fatigue (B = 0.66, p <0.001), SNS fatigue positively predicted irrational procrastination (B = 0.10, p <0.001), and SNS addiction positively predicted irrational procrastination(B = 0.14, p <0.001). Furthermore, we used the Sobel test to examine the significance of the indirect effect of SNS fatigue in the relationship between SNS addiction and irrational procrastination. The results indicated that SNS fatigue significantly mediated the relationship between SNS addiction and irrational procrastination (z = 7.97, p <0.001).

Additionally, there was a significant SNS addiction× effortful control interaction effect on SNS fatigue (B = -0.26, p <0.001) in mediator variable model. In dependent variable model, there was a significant SNS addiction × effortful control interaction effect (B = -0.07, p <0.05) and a significant SNS fatigue × effortful control interaction effect on irrational procrastination (B = -0.08, p <0.001). These findings indicated that the association between SNS addiction and irrational procrastination, the association between SNS addiction and SNS fatigue, and the association between SNS fatigue and irrational procrastination were all moderated by effortful control.

**Fig 2:**
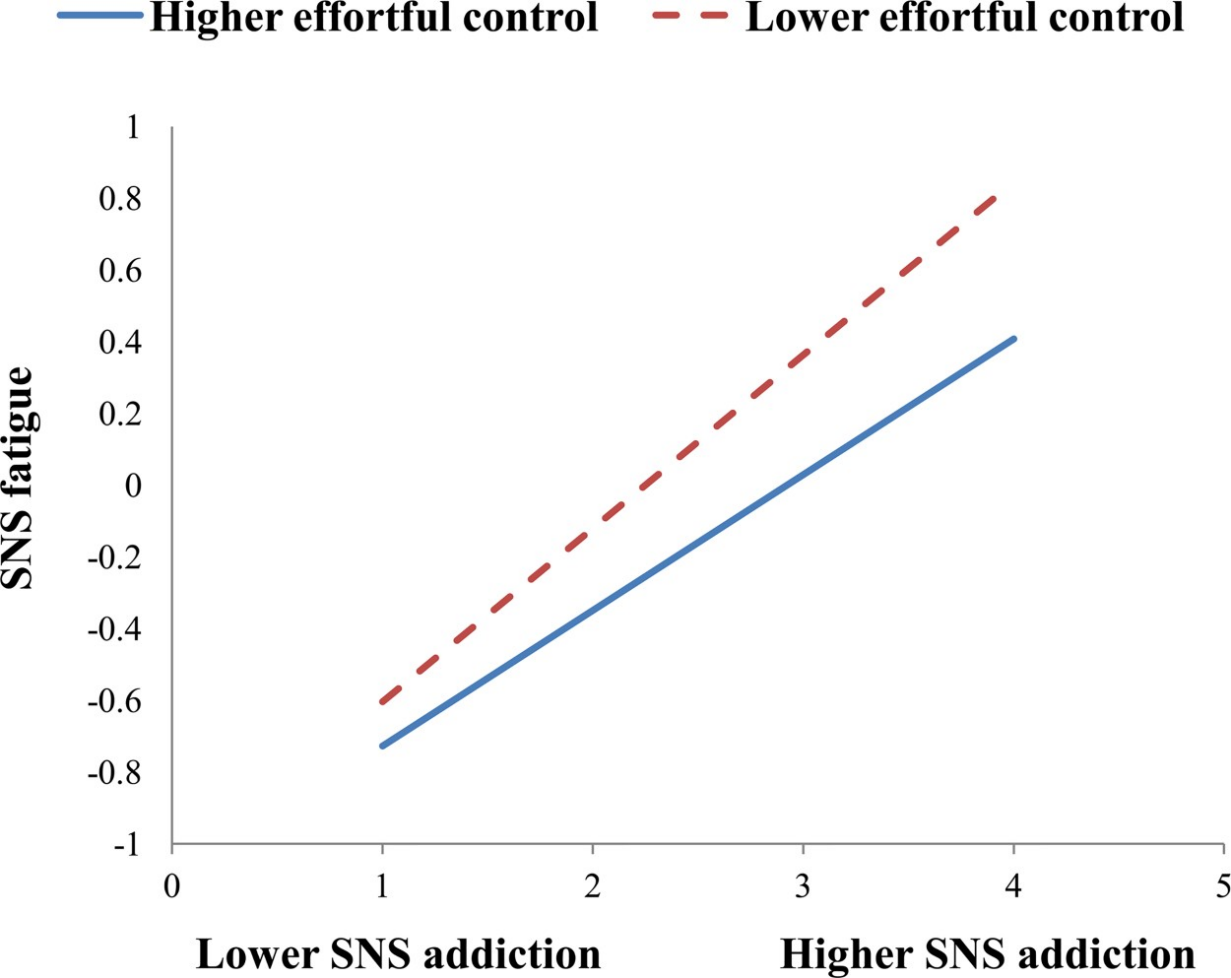
Effortful control moderates the relation between SNS addiction and SNS fatigue.

**Fig 3:**
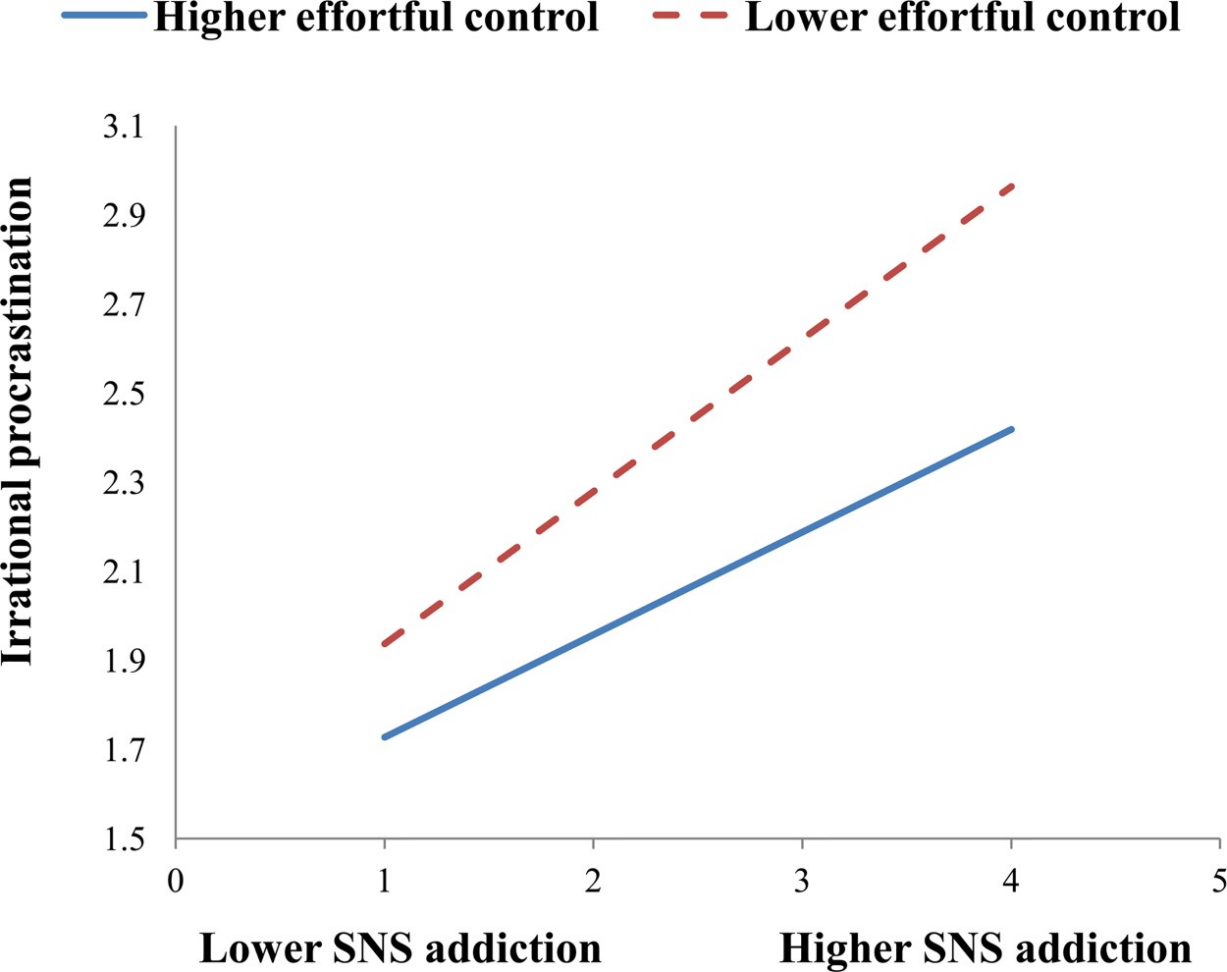
Effortful control moderates the relation between SNS addiction and irrational procrastination.

**Fig 4:**
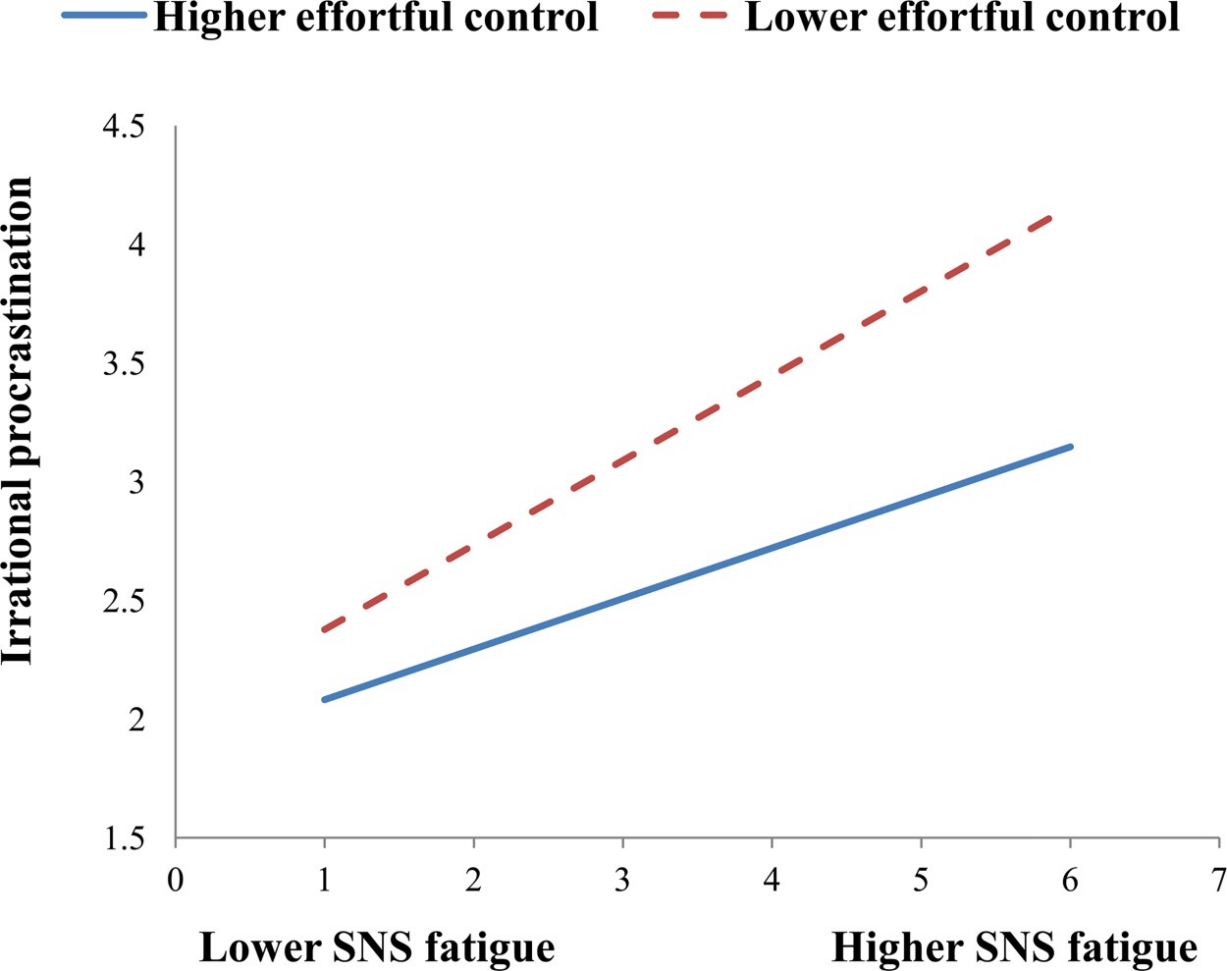
Effortful control moderates the relation between SNS fatigue and irrational procrastination.

To illustrate these interactions, we also performed simple slope analyses to explore whether slopes for the high-effortful control group (1 SD above the mean) were different from slopes for the low-effortful control group (1 SD below the mean) in the two models. As shown in Fig 2, the effect of SNS addiction on SNS fatigue was stronger for the low-effortful control group (B = 0.48, t = 13.86, p<0.001) than for the high-effortful control group (B = 0.38, t = 10.86, p<0.001). As shown in Fig 3, the effect of SNS addiction on irrational procrastination was stronger for the low-effortful control group (B = 0.34, t = 10.49, p<0.001) than for the high-effortful control group (B = 0.23, t = 7.93, p<0.001). As shown in Fig 4, the effect of SNS fatigue on irrational procrastination was stronger for the low-effortful control group (B = 0.36, t = 11.19, p<0.001) than for the high-effortful control group (B = 0.21, t = 6.68, p<0.001).

Furthermore, the two conditional analyses indicated that all of the conditional direct effects and indirect effects were positively and significantly different from zero. Namely, both the direct effect of SNS addiction on irrational procrastination and the indirect effect of SNS addiction on irrational procrastination through SNS fatigue were stronger for the lower-effortful control group.

## Discussion

Although studies have begun to illuminate the relationship between SNS use and procrastination [7], none has investigated how (or why), and when (i.e., under what conditions) SNS addiction leads to irrational procrastination. To fill this gap, the present study conducted a moderated mediation model, in which SNS fatigue is included as a mediator and effortful control as a moderator. The results indicated that SNS addiction was positively associated with irrational procrastination, hypothesis 1a was supported. Undergraduate students’ SNS fatigue mediated the relation between SNS addiction and irrational procrastination, hypothesis 1b was supported. Effortful control played an important role as a moderator. Specifically, the direct association between SNS addiction and irrational procrastination was stronger for students with low effortful control, as was the mediating effect of SNS fatigue, hypothesis 2 and hypothesis 3 were supported. These results generate explanations of factors that carry the influence of SNS addiction on to irrational procrastination (mediation) and of contextual factors on which this influence depends (moderation). In other words, these findings have implications for understanding how SNS addiction is linked to irrational procrastination, and for whom this influence is strongest.

Firstly, our study found that SNS addiction could significantly predict irrational procrastination in undergraduate students. This result is consistent with a previous study indicating the effect of SNS use (SNS checking and SNS enjoyment) on procrastination [7]. SNS addicts motivated by social interaction and communication check their SNS accounts and communicate with SNS friends in an automatic and impulsive fashion [24, 62]. According to Davis’s cognitive–behavioral theory [63], individuals experiencing SNS addiction may spend more time and cognitive resources on SNS use, accompanying a growing neglect of offline professional, social, and personal responsibilities, which results in negative consequences, such as irrational procrastination.

In addition, communication overload caused by SNS addiction may interrupt users’ daily tasks [26, 64], making it harder to concentrate and easier to discontinue activities at hand [7, 26]. Moreover, because of being perceived as stressful, frustrating, or boring, tasks on which students procrastinate (e.g., writing an academic paper) may increase short-term negative affect during task engagement [65]. Therefore, undergraduate students suffering from SNS addiction are more likely to engage in activities on SNS, such as checking SNS and passing time on SNS, which may induce irrational procrastination [7]. Social networking, therefore, has been seen as a particularly prominent source of procrastination among students [66].

Secondly, our study indicated that SNS fatigue is an important, underlying psychosocial mechanism in the relation between SNS addiction and irrational procrastination. SNS addiction may lead to a subjective feeling of tiredness from SNS usage (i.e., SNS fatigue), which in turn may result in irrational procrastination. This finding was congruent with the self-regulation failure theory and previous studies arguing that irrational procrastination, as self-regulatory failure, was caused by fatigue, a depletion of resources and a lowered capacity for adequate self-regulation [29, 30]. Students experiencing SNS addiction are often flooded by information on SNS, such as information about the personal lives, news, expertise, and gossip, exceeding their information processing capacity and resulting in information overload [67]. Information overload has been proved to have positive relationship with SNS fatigue [26].

In addition to experiencing information overload, students experiencing SNS addiction can also experience social overload or social interaction overload [67, 68]. When the demands of on-line social interactions extend beyond their communicative and cooperative capability, SNS users will become overwhelmed and thereby fatigued [26]. Given that fatigue refers to a subjective feeling of decreased motivation and task aversion, it can be considered as an important predictor of procrastination [29]. Our study concluded that SNS fatigue mediated the association between SNS addiction and irrational procrastination. It is noteworthy that the relationship between SNS addiction and irrational procrastination also could be mediated by other potential valuables, such anxiety and stress. SNS addiction or SNS use has been shown to be positively associated with various mental health problems, such as symptoms of depression, anxiety, and stress [25, 69]. Anxiety and depression have been proved to be the causes of procrastination [70]. In other words, the relationship between SNS addiction and undergraduate students' irrational procrastination also could be mediated by anxiety and depression. Therefore, it's not appropriate for us to put forward the full mediation based on a specific mediating model containing only one mediator variable. Above all, our study further concluded that SNS fatigue played a partially mediating role in the relation between SNS addiction and undergraduate students' irrational procrastination.

The most important finding of the current study was that both the direct effect that SNS addiction itself exerted on irrational procrastination and the indirect effect that SNS addiction exerted on irrational procrastination via SNS fatigue were moderated by effortful control, with these effects being stronger for students with lower effortful control. Specifically, effortful control not only attenuates the effects of SNS addiction on irrational procrastination, but also mitigates the indirect impact of SNS addiction on irrational procrastination through the mediating role of SNS fatigue. In addition, both the association between SNS addiction and SNS fatigue and the relation between SNS fatigue and irrational procrastination were moderated by effortful control. These findings were in accordance with the risk-buffering hypothesis, which states that effortful control, as a favorable individual characteristic, can buffer the potential negative effects of risk factors on individuals’ emotional and behavioral problems [47, 48, 51].

Specifically, effortful control could have a moderating effect in the link between SNS addiction and students’ behavioral problems (irrational procrastination) through cognitive and motivational mechanisms. In terms of cognitive mechanisms, individuals with high effortful control may be able to adjust the direction and duration of their attention and inhibit prepotent behavior (such as checking SNS), as well as shift their attention to tasks that have been put off (e.g., writing an academic paper) to meet situational demands [71, 72]. This perspective is in line with the self-regulatory failure model [20, 45]. In terms of the motivational mechanisms, individuals with high effortful control are thought to be better at initiating, sustaining, and regulating their motivation and engaging in goal-directed activities, such as academic tasks [44, 73]. Thus, students with higher effortful control might be able to inhibit the temptation of immediate gratifications from SNS usage, such as the satisfaction of having relatedness needs met online, and to maintain their achievement motivation. The effect of SNS addiction on irrational procrastination may thus be mitigated.

Both the moderating effect of effortful control in the association between SNS addiction and SNS fatigue and the moderating effect of effortful control in the relation between SNS fatigue and irrational procrastination may be explained by the emotional regulation mechanism of effortful control. Individuals with high effortful control are expected to be more successful at coping with or eliminating their negative emotions and reducing the potential unwanted effect of SNS use on their subjective feelings [41, 42]. They are often thought to have well-developed endurance, willpower and resilience [74, 75]. Therefore, even if students with high effortful control feel tired from overuse of SNS, they may nevertheless devote themselves to their duties. Thus, fatigue caused by the SNS addiction may be relieved by the well-developed emotional management skills of students with high effortful control.

## Limitations and implications

Although our study provides novel insights into the association between SNS addiction and irrational procrastination, some limitations should be taken into account when interpreting the results. The present study was limited by its cross-sectional design, and future researchers can adopt longitudinal and experimental methods to examine the direction of the proposed relationships. Firstly, future researchers can test the causal relationships among SNS addiction, SNS fatigue and irrational procrastination. Secondly, since effortful control may help prevent people from being addicted to SNS addiction, future researchers may also test the causal relationships between SNS addiction and effortful control. In addition, the validity of the self-report data may be restricted by social desirability bias. Multi-rater assessment should be adopted in future research to collect information not just from students, but also from parents, teachers and peers. Generalization is also a concern, as the proposed model was tested in a sample of undergraduate students from two universities in China; we are not certain how well our results would generalize to adolescents, wage-earners and other populations from different cultural backgrounds. Moreover, although all of the data were collected in classrooms, the class information of the participants had not been recorded. To explore the effect of class atmosphere on undergraduate students and make data analysis more scientific and precise, intra class correlation should be controlled in the process of data analysis in future research.

Our findings have important practical implications for both undergraduate students and educators. They can offer constructive suggestions for undergraduate students to reduce the occurrence of the irrational procrastination caused by excessive use of SNS. Given that fatigue from SNS usage is a significant bridge linking SNS addiction and irrational procrastination, undergraduate students suffering from SNS addiction (especially those with lower effortful control) could consciously reduce time on SNS to rescue them from feelings of fatigue and irrational procrastination in dealing with their main task. They also could prevent irrational procrastination through relaxation after a long period of catching up on SNS. A prior study illustrated that listening to relaxing music alleviated the mental fatigue associated with performing an enduring cognitive-motor task [76]. Our findings can also offer effective practical guidance for educators to develop targeted intervention strategies to promote students' adaptability to society. Firstly, educators should warn undergraduate students that SNS addiction may act as a trigger for irrational procrastination and guide them in moderate SNS use. Secondly, effortful control appears not only to alleviate the effect of SNS addiction on SNS fatigue, but also to alleviate the effect of SNS fatigue on irrational procrastination. According to the findings of a prior study, effortful control can be improved by intervention programs and daily practice, despite the fact that it has been seen as a relatively stable individual characteristic [77]. Inzlicht and his colleagues stated that people can improve their effortful control by setting better and more self-aligned goals in the early days; effortful control also can be established and improved by paying attention to the discrepancies between goals and current behavior; and effortful control can be established by cultivating people’s capacity of monitoring the conflicts between goals and actual behavior. Therefore, educators could help undergraduate students suffering from SNS addiction avoid the feelings of fatigue and irrational procrastination caused by excessive use of SNS by improving their effortful control.

This study, to our knowledge, is the first to examine the association between SNS addiction and irrational procrastination by focusing on the mediating and moderating processes affecting this association in undergraduate students. Effortful control and SNS fatigue played important roles in predicting what kind of students with SNS addiction would engage in irrational procrastination.

Future research should focus on possible reciprocal processes in the associations identified in the current study. Specifically, additional researches also needed to consider other potential mediators (such as sleep quality) and moderators (such as mindfulness) to reveal much more plausible argument how (or why) and when SNS addiction leads to neglecting other duties. For example, sleep quality has been demonstrated to mediate the link between social media use and cognitive failure [36], which was positively correlated with procrastination [78]. Therefore, the association between SNS addiction and irrational procrastination may also be mediated by sleep quality. Mindfulness, as an individual characteristic, played a moderating role in the relation between mobile phone addiction and sleep quality [58]. Thus, mindfulness may moderate the relation between SNS addiction and irrational procrastination, with the relation being stronger for students with lower mindfulness.

## Supporting information

S1 Dataset Dataset used for analyses in present study. (SAV).
(ZIP)

S1 Measurements Measurements used in present study. (DOCX)
