# Supplementary material for: Social networking site addiction and undergraduate students’ irrational procrastination: The mediating role of social networking site fatigue and the moderating role of effortful control
Source: PLoS One. 2018 Dec 11;13(12):e0208162. doi: 10.1371/journal.pone.0208162 (PMC6289504; doi:10.1371/journal.pone.0208162)
Supplement: S1 Measurements — (DOCX) [file pone.0208162.s002.docx]

**同学们好，本次问卷调查为匿名调查，请放心作答。你的答案没有对错之分，按照自身情况填写完整即可。非常感谢你的参与！本问卷中，社交网站是指QQ空间，微信朋友圈或人人网等基于互联网的社会交往服务平台，请根据你最常使用的社交网站作答。**

1. 你的性别：男 or 女 2. 你的年龄：

3. 你的年级：大一 大二 大三 大四

4.**请在下面每个句子右边的评价值一栏中，在最符合你真实情况的数值上标记。**

|  | **非常不同意** | **比较不同意** | **不确定** | **比较同意** | **非常同意** |
| --- | --- | --- | --- | --- | --- |
| 1.我把事情拖得太久，以至于我的幸福感或效率受到不必要的损害。 | 1 | 2 | 3 | 4 | 5 |
| 2.如果有一件我应该做的事情，我会先完成这件事再去做其他不重要的事 | 1 | 2 | 3 | 4 | 5 |
| 3.如果我能早点做一些事情，我的生活会更好 | 1 | 2 | 3 | 4 | 5 |
| 4.当我应该做一件事的时候，我会做另一件事 | 1 | 2 | 3 | 4 | 5 |
| 5.到了晚上，我意识到我可以更好地度过一天的时间 | 1 | 2 | 3 | 4 | 5 |
| 6.我会合理规划我的时间 | 1 | 2 | 3 | 4 | 5 |
| 7.我会毫无道理地延迟工作 | 1 | 2 | 3 | 4 | 5 |
| 8.我会拖延 | 1 | 2 | 3 | 4 | 5 |
| 9.无论何事，当我认为需要做的时候，我就会去做 | 1 | 2 | 3 | 4 | 5 |

**5.请在下面每个句子右边的评价值一栏中，在最符合你真实情况的数值上标记。**

|  | **完全**  **不同意** | **比较**  **不同意** | **有点**  **不同意** | **不**  **确定** | **有点同意** | **比较同意** | **完全同意** |
| --- | --- | --- | --- | --- | --- | --- | --- |
| 1.持续使用社交网站后我感到很难放松。 | 1 | 2 | 3 | 4 | 5 | 6 | 7 |
| 2.使用社交网站之后我感到疲倦。 | 1 | 2 | 3 | 4 | 5 | 6 | 7 |
| 3.由于使用社交网站的原因，我感到相当疲惫。 | 1 | 2 | 3 | 4 | 5 | 6 | 7 |
| 4.我在空闲时间使用社交网站之后，需要努力才能集中精力。 | 1 | 2 | 3 | 4 | 5 | 6 | 7 |
| 5.使用社交网站的时候，我感到过于疲倦以致不能很好地完成其他任务。 | 1 | 2 | 3 | 4 | 5 | 6 | 7 |

**6.请就以下陈述符合你实际情况的程度做出评定。**

|  | **完全不符合** | **比较不符合** | **不确定** | **比较符合** | **完全符合** |
| --- | --- | --- | --- | --- | --- |
| 1.社交网站使用让我无法专心学习。 | 1 | 2 | 3 | 4 | 5 |
| 2.每天一早醒来，我最先想到的事就是登陆社交网站。 | 1 | 2 | 3 | 4 | 5 |
| 3.社交网站使用过多，让我难以入睡。 | 1 | 2 | 3 | 4 | 5 |
| 4.社交网站使用干扰了我的日常社交活动。 | 1 | 2 | 3 | 4 | 5 |
| 5.我的亲朋好友认为我的社交网站使用时间过多。 | 1 | 2 | 3 | 4 | 5 |
| 6.无法正常使用社交网站，让我感到焦躁不安。 | 1 | 2 | 3 | 4 | 5 |
| 7.情绪低落时，我常登陆社交网站以使自己好受点。 | 1 | 2 | 3 | 4 | 5 |
| 8.我曾试图减少社交网站使用时间，但最终并没做到。 | 1 | 2 | 3 | 4 | 5 |

**7.请在下面每个句子右边的评价值一栏中，在最符合你真实情况的数值上标记。**

|  | **完全不符合** | **比较不符合** | **有些不符合** | **有些符合** | **比较符合** | **完全符合** |
| --- | --- | --- | --- | --- | --- | --- |
| 1. 有时我明知道不该做某事，但最终还是做了。 | 1 | 2 | 3 | 4 | 5 | 6 |
| 2. 即使任务很困难，我也会按时完成。 | 1 | 2 | 3 | 4 | 5 | 6 |
| 3. 晚上进入家里漆黑的房间时，我感到害怕。 | 1 | 2 | 3 | 4 | 5 | 6 |
| 4. 我越想阻止自己做某些不该做的事，就越有可能去做。 | 1 | 2 | 3 | 4 | 5 | 6 |
| 5. 即使不喜欢某门课，但为了取得好成绩我也会努力学习。 | 1 | 2 | 3 | 4 | 5 | 6 |
| 6. 我害怕一个人独自呆在家里。 | 1 | 2 | 3 | 4 | 5 | 6 |
| 7. 明知不应该，我还是会在做作业之前先玩一会。 | 1 | 2 | 3 | 4 | 5 | 6 |
| 8. 即使对某门课不感兴趣，我也会认真听讲。 | 1 | 2 | 3 | 4 | 5 | 6 |
| 9. 我担心会有不好的事情发生在自己身上。 | 1 | 2 | 3 | 4 | 5 | 6 |
| 10. 我不能很好地抵制诱惑。 | 1 | 2 | 3 | 4 | 5 | 6 |
| 11. 我能在规定时间内完成作业。 | 1 | 2 | 3 | 4 | 5 | 6 |
| 12. 我担心自己会惹上什么麻烦。 | 1 | 2 | 3 | 4 | 5 | 6 |
| 13. 我会做一些让自己快乐但可能对自己有害的事情。 | 1 | 2 | 3 | 4 | 5 | 6 |
| 14. 如果有困难的任务需要完成，我会马上动手做。 | 1 | 2 | 3 | 4 | 5 | 6 |
| 15. 我害怕独自走夜路。 | 1 | 2 | 3 | 4 | 5 | 6 |
| 16. 我比大多数人更胆小。 | 1 | 2 | 3 | 4 | 5 | 6 |
